# Rapamycin ameliorates chronic intermittent hypoxia and sleep deprivation-induced renal damage via the mammalian target of rapamycin (mTOR)/NOD-like receptor protein 3 (NLRP3) signaling pathway

**DOI:** 10.1080/21655979.2022.2037872

**Published:** 2022-02-19

**Authors:** Wei Liu, Dong Zhao, Xiaofeng Wu, Fang Yue, Haizhen Yang, Ke Hu

**Affiliations:** Department of Respiratory and Critical Care Medicine, Renmin Hospital of Wuhan University, Wuhan, Hubei, China

**Keywords:** Rapamycin, chronic intermittent hypoxia, sleep deprivation, renal damage, mTOR, NLRP3

## Abstract

Rapamycin inhibits the activation of NOD-like receptor protein 3 (NLRP3) by regulating the mammalian target of rapamycin (mTOR) to treat obstructive sleep apnea-related renal injury. Sleep deprivation (SD) and chronic intermittent hypoxia (CIH) mouse models were used to assess the effects of autophagy in vivo. Compared with the control, SD, and CIH groups, the SD+CIH group had lower body weight and higher levels of blood urea nitrogen (BUN), creatinine, and urinary albumin (U-Alb) (P < 0.05); renal injury and oxidative damage occurred in the SD+CIH group, the kidney cell nucleus ruptured, and morphological structure of the cells was unclear in the SD+CIH group. The SD+CIH group demonstrated increased apoptosis compared with the control, SD, and CIH groups using Western blot analysis. Compared to the control, SD, and CIH groups, the SD+CIH group showed a higher degree of microtubule-associated protein light chain 3\ staining. Compared to the SD+CIH group, BUN, creatinine, and U-Alb levels decreased, and apoptosis increased in the SD+CIH+rapamycin group, and the structure of the kidney after rapamycin treatment was well preserved. The mTOR expression was increased in the kidneys of the SD+CIH group. The NLRP3, Gasdermin D (GMDSD), interleukin (IL)-18, IL-1β, and cleaved-caspase-1 protein levels were higher in the SD+CIH group than the SD+CIH+rapamycin group, and the NLRP3, GMDSD, IL-18, IL-1β, and cleaved-caspase-1 mRNA levels were higher in the SD+CIH group than the SD+CIH+rapamycin group. Following rapamycin treatment, pyroptosis was suppressed. Rapamycin ameliorates renal damage by inhibiting the mTOR/NLRP3 signaling pathway.

## Graphical abstract

**Figure:**
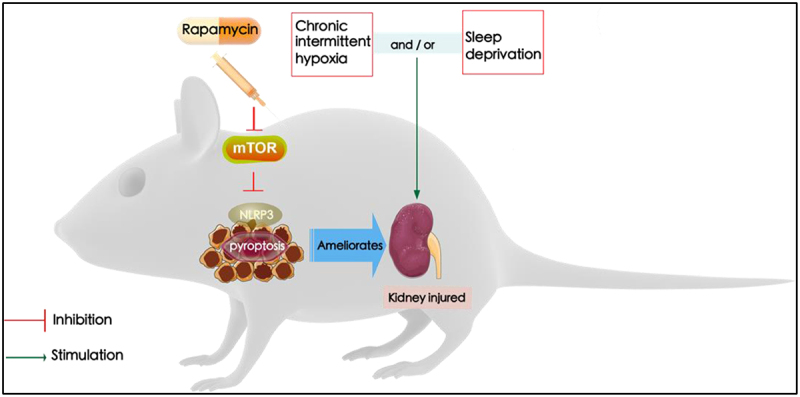

## Introduction

Obstructive sleep apnea (OSA) is the most common sleep-disordered breathing disease. Epidemiological studies have shown that the incidence rate of adults is 9–38% [1]. OSA is characterized by recurrent upper airway collapse, occlusion leading to OSA or hypopnea, chronic intermittent hypoxia (CIH), and sleep deprivation (SD). During intermittent hypoxia, the body produces a large number of oxygen free radicals after repeated hypoxia, resulting in damage to various organs. Respiratory awakening leads to the disintegration of normal sleep structure, SD, and a vicious cycle [2]. SD refers to a lack of sleep or a serious lack of sleep time for various reasons. SD can lead to serious health challenges [3], triggering many short and long-term complications and negative effects [4]. SD leads to physiological clock disorders, shortened sleep time, and reduced sleep depth and continuity. SD affects the systemic inflammatory response, causes an imbalance in antioxidant oxidation products, affects the production and metabolism of a variety of cytokines, and aggravates the process of intermittent hypoxia. The pharyngeal airway of patients with OSA collapses during sleep, resulting in hypoxia and termination after awakening.

Recent studies have shown that OSA may increase the incidence of chronic kidney disease [5] and acute kidney injury [6]. The kidneys are not only regulated by activities, diet, temperature, and the endocrine system but is also regulated by circadian genes. Long-time interference with sleep time in rats leads to peroxidation damage in renal tissue. Wisit et al. found that people with short sleep durations were more prone to proteinuria [7]. Intermittent hypoxia induces oxidative stress and activates inflammatory factors. Studies have found that mice with SD often experience multiple organ inflammatory reactions [8]. The kidney is rich in mitochondria, and the mitochondrial respiratory chain and nicotinamide adenine dinucleotide phosphate oxidase are the main sources of reactive oxygen species (ROS), which are considered to be important factors leading to cardiovascular disease and diabetes. These diseases can also lead to acute kidney injury [9], which indicates that the kidneys are highly susceptible to oxidative stress injury [10]. Continuous positive airway pressure is the main therapy for OSA [11]; however, there is no good way to deal with renal injury caused by OSA. Autophagy refers to the degradation of the cytoplasmic components of the lysozyme. Generally, autophagy is an important protective mechanism of the body, and enhancing the autophagy process can appropriately reduce the oxidative damage of the body; however, over-activated autophagy will also lead to cell death and organ damage. Yang et al. found that oxidative stress can mediate the change in mammalian target of rapamycin (mTOR), an important target of autophagy [12]. During the occurrence of renal injury, the level of intracellular autophagy increases to a certain extent with the aggravation of oxidative stress injury. The NOD-like receptor protein 3 (NLRP3) inflammasome is an important target of the classical focal death pathway. The level of NLRP3 is significantly increased in patients with OSA [13], mTOR is an important target mediating autophagy, inhibition of the mTOR signaling pathway can significantly reduce the level of NLRP3 and reduce organ damage caused by OSA [14], and the mTOR/NLRP3 pathway can be used as a potential intervention target to increase autophagy and then reduce cell death to delay renal injury [15].

Rapamycin, an autophagy activator, is involved in various cellular processes by inhibiting mTOR, such as protein translation, ribosome biogenesis, mitochondrial oxygen consumption, proliferation, and differentiation [16]. Rapamycin modulates multiple cellular signaling pathways in OSA. a can improve renal function, decrease proteinuria levels, and alleviate renal tubulointerstitial infiltration, and interstitial fibrosis [17]. In the mesangial proliferative rat model, rapamycin significantly reduced various symptoms of glomerulonephritis [18]. Therefore, we speculate that rapamycin may play an important role in the prevention and treatment of nephropathy through its anti-inflammatory and anti-proliferative effects. However, studies on the influence of rapamycin on OSA-induced renal injury in vivo are limited.

Although the evidence demonstrates that OSA-induced pyroptosis occurs when oxidative stress is overproduced, the mechanism of pyroptosis leading to an inflammatory response in OSA-related renal injury remains unknown. This study aimed to explore whether rapamycin inhibits the activation of NLRP3-dependent pyroptosis by regulating mTOR, to reduce OSA-related renal injury, and to provide a new basis for the diagnosis and treatment of OSA-related renal injury.

## Materials and methods

### Mouse model experimental protocol

Female C57BL/6 J mice (6–8 weeks old) were randomly allocated into the following six groups (six mice in each group): control, SD, CIH, SD+CIH, rapamycin, and SD+CIH+rapamycin groups. The SD+intermittent hypoxia device used was a rectangular cabin (24.5 × 21.0 × 19.7 cm) made of 4-mm-thick plexiglass (Biospherix, USA, A84XOV). The cabin was equipped with sleep deprivation-related equipment, such as a metal sweeping rod, electric motor, and frequency modulation knob (1 min/cycle). The metal scanning rod sweeps from one side of the box to the other side to provide the mouse with relatively mild intermittent tactile stimulation. The animals in the SD groups were deprived of sleep for 18 h (4:00 PM to 10:00 AM). Mice can be prevented by gently crossing the rod. Simultaneously, two holes with a diameter of 5 mm were placed in the center of the sidewall of the cabin to input hypoxic gas and air to prepare the hypoxic animal cabin. The mice were exposed to intermittent hypoxia for 8 h/day (08:00 AM to 4:00 PM) [19]. Oxygen concentrations in the chamber were monitored using a heated zirconium sensor connected to solenoid valves that controlled the flow of oxygen and nitrogen. The valves were operated using a microprocessor-controlled timer. The mice were exposed to 30s of 5% inspired oxygen every 2 min. Control mice were housed under normoxic conditions, similar to the chamber. Mice were acclimated for 6 days before starting the experiments, and on the 7th day, the mice in each group were placed in a SD combined with intermittent hypoxia chamber to adapt to the experimental environment for 24 h, and the chamber started operating on days 8–28. In the SD+CIH group, 18 h (4:00 PM to 10:00 AM) of SD and 8 h (8:00–16:00) of intermittent hypoxia were performed every day [20]. In all rapamycin groups, mice received an intraperitoneal injection of rapamycin (MedChemExpress, USA) at a concentration of 4 mg/kg on days 8–28 (once/day). After the study, the mice were sacrificed under general anesthesia; serum and urine [21] were collected; and the left kidney tissue was excised for the following experiment.

### Histological assessments

The harvested kidneys were fixed in 4% paraformaldehyde for 24 h. The sections were then embedded in paraffin, cut into sections of 5-μm thickness, and stained with hematoxylin and eosin (HE) [22]. Histological assessment was conducted using a light microscope (Olympus, Tokyo, Japan).

Paraffin sections were dewaxed, hydrated, and rinsed with distilled water. According to the requirements of the first antibody to tissue antigen repair, the slices were dripped with endogenous peroxidase blocker and rinsed with phosphate-buffered saline (PBS), then the first antibody was dropped on the section, and after incubated at 25°C and rinsed with PBS; primary antibody enhancer (Proteintech, Wuhan, China) was added to the section and incubated at room temperature, then washed with PBS; and secondary antibody-horseradish peroxidase (HRP) polymer (Proteintech, Wuhan, China) reagent was added, incubated at room temperature, and washed. The following antibodies were used: LC3 (Abmart, PA9415); mTOR (Abmart, T55306); and NLRP3 (Cell Signaling Technology, 15101).

### Oxidative stress analysis

Commercial kits for detection of malondialdehyde (MDA), superoxide dismutase (SOD), catalase (CAT), and glutathione (GSH) were obtained from Nanjing Jiancheng Bioengineering Institute (Nanjing, China). The levels of MDA (A003-2) and CAT (A007-1-1) were monitored by measuring the absorbance at 532 nm and 240 nm, respectively. The enzymatic activities of SOD (A001-3) and GSH (A006-2-1) were measured at 450 and 405 nm, respectively.

### Biochemical assays

Blood urea nitrogen (BUN), creatinine, and urinary albumin (U-Alb) contents were measured using the respective kits according to the manufacturer’s instructions. BUN (C013-2-1), creatinine (C011-2-1), and urine microalbumin assay kits (E038-1-1) were purchased from Nanjing Jiancheng Institute of Biotechnology (Nanjing, China). BUN, creatinine, and U-Alb levels were measured at 640 nm, 546 nm, and 340 nm, respectively.

### RNA isolation and quantitative real-time PCR

Total RNA from mouse kidneys was isolated using TRIzol (Invitrogen), and reverse transcription was performed using 500 ng of total RNA in the first-strand cDNA synthesis reaction with PrimeScript RT reagent Kit (Takara). The expression of each gene was determined using an ABI Prism 7500 PCR system (Applied Biosystems). The reaction conditions were as follows: pre-denaturation stage: 95°C for 30s, 1 cycle; amplification: 95°C for 5s, 60°C for 30s, 40 cycles; and dissolution curve analysis stage: 95°C for 15s, 60°C for 1 min, and 95°C for 15s. The primer sequences (forward and reverse) used in this study were as follows: NLRP3 (forward: CGTGAGTCCCATTAAGATGGAGT, reverse: CCCGACAGTGGATATAGAACAGA); GSDMD (forward: GTGTGTCAACCTGTCTATCAAGG, reverse: CATGGCATCGTAGAAGTGGAAG); Caspase-1 (forward: TTTCCGCAAGGTTCGATTTTCA, reverse: GGCATCTGCGCTCTACCATC); IL-18 (forward: TCTTCATTGACCAAGGAAATCGG, reverse: TCCGGGGTGCATTATCTCTAC); and IL-1β (forward: AGCTACGAATCTCCGACCAC, reverse: CGTTATCCCATGTGTCGAAGAA). The gene expression was determined using the 2− ΔΔCT method.

### Western blotting

Approximately 30 mg of mouse kidney tissue was homogenized, the total protein was extracted using RIPA buffer, and the protein concentration was determined using a total protein assay kit. NLRP3, Gasdermin D (GMDSD), cleaved-caspase-1, interleukin IL)-18, IL-1β, microtubule-associated protein light chain 3 (LC3), sequestosome 1 (P62), Beclin 1, mTOR, and phospho-mammalian target of rapamycin (p-mTOR) protein was measured by Western blotting using monoclonal mouse anti-human NLRP3 (Cell Signaling Technology, 15101) and rabbit anti-human GMDSD (Cell Signaling Technology, 97558), cleaved-caspase-1 (Cell Signaling Technology, 4199), IL-18 (Cell Signaling Technology, 57058), IL-1β (Cell Signaling Technology, 12703), LC3 (Cell Signaling Technology, 12741), P62 (Cell Signaling Technology, 16177), Beclin 1 (Cell Signaling Technology, 3495), mTOR (Cell Signaling Technology, 2983), and p-mTOR (Cell Signaling Technology, 5536). The proteins were separated on 10% polyacrylamide gels and transferred onto polyvinylidene difluoride membranes, primary antibody (1:1000) at 4°C overnight with gentle shaking and incubated with an appropriate HRP-labeled secondary antibody for 90 min at room temperature on a shaker. Finally, the membranes were again washed three times with 1× TBST and assayed using a ChemiDoc XRS gel imaging system (Bio-Rad, Hercules, CA, USA). The protein expression levels were quantified using Image J software (NIH Image, Bethesda, MD, USA).

### Transmission electron microscopy

Kidney tissues were fixed in 2.5% glutaraldehyde and 4% paraformaldehyde dissolved in 100 mM sodium phosphate (pH 7.2) [23]. The tissues were washed using 100 mM sodium cacodylate (pH = 7.4), fixed in 2% osmium tetroxide, and washed again. The fixed tissues were dehydrated across an ethanol gradient and propylene oxide and then embedded in epoxy resin (Tab 812 Resin; Canemco Inc., Montreal, QC, Canada). The resulting ultrathin (60–70 nm) sections were counterstained with uranyl acetate and lead citrate and then viewed using a Hitachi 7600 transmission electron microscope (Hitachi High-Technologies America, Inc., Schaumburg, IL, USA) equipped with a MacroFire monochrome progressive scan CCD camera (Optronics, 10 Inc., Muskogee, OK, USA) with AMTv image capture software (Advanced Microscopy Techniques, Inc., Danvers, MA, USA).

### Statistical analysis

Data are expressed as mean ± standard deviation. All statistical analyses were performed using GraphPad Prism 6.0 (GraphPad Software, La Jolla, CA, USA). A two-tailed paired Student’s t-test was used for comparison between two groups and one-way analysis of variance with Tukey’s post hoc test for more than two groups. A p-value < 0.05 was considered as an indicator of a statistically significant difference.

## Results

### Weight and changes in renal function in chronic intermittent hypoxia and sleep-deprived mice

OSA is characterized by CIH and SD and can lead to kidney injury. The study aimed to explore whether the mechanism of rapamycin inhibits the activation of NLRP3 inflammatory bodies by regulating mTOR, to explore the therapy of OSA-induced kidney injury.

**Figure 1.**
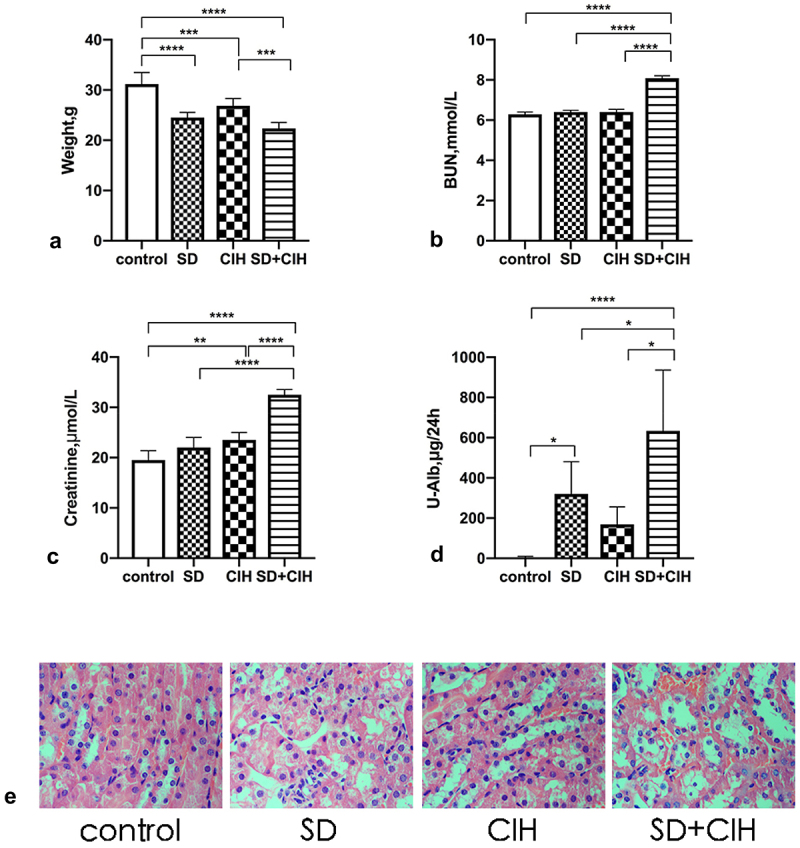
Weight and changes in renal function in chronic intermittent hypoxia and sleep-deprived mice. (a) Mouse weight (grams); (b) Blood urea nitrogen (BUN); (c) Creatinine; (d) Urinary albumin (U-Alb); (e) Representative images of hematoxylin and eosin (HE)-stained kidney tissues in four different groups (original magnification, × 40). *p < 0.05; **p < 0.01; ***p < 0.001; ****p < 0.0001.

Compared with the control group, the SD, CIH, and SD+CIH groups had lower body weights, and the difference was statistically significant (P < 0.05) (Figure 1(a)). Compared with the control, SD, and CIH groups, the SD+CIH group had higher BUN, creatinine, and U-Alb levels, and the difference was statistically significant (P < 0.05) (Figure 1(b-d)). The obvious renal injury occurred in the CIH + SD group in images of HE-stained kidney tissues, including inflammatory cell infiltration, nucleus abscission, unclear cell boundary, and tissue edema. Renal injury in the SD and CIH groups was between the control group and the CIH + SD group (Figure 1(e)).

### Effect of chronic intermittent hypoxia and sleep deprivation on the antioxidant status

**Figure 2.**
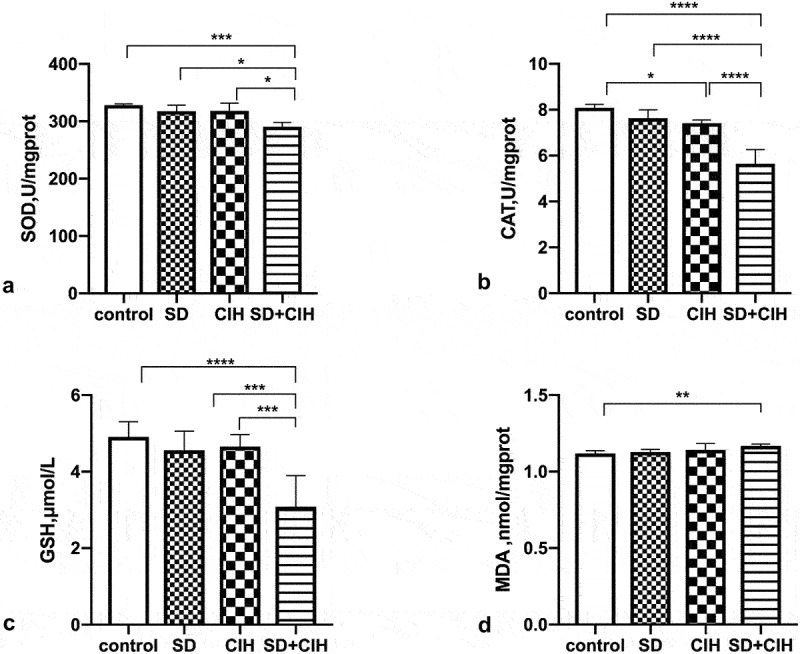
Effect of chronic intermittent hypoxia and sleep deprivation on the antioxidant status. (a) Calculation of malondialdehyde (MDA) abundances. (B, C, D) Calculation of superoxide dismutase (SOD), catalase (CAT), and glutathione (GSH) activities. *p < 0.05; **p < 0.01; ***p < 0.001; ****p < 0.0001.

Accumulating evidence indicates that CIH/SD contributes to oxidative stress and inflammation. Therefore, we investigated the effect of SD/CIH on the activation of these processes. Figure 2(a-d) show that CIH+SD exposure has induced oxidative damage, as evidenced by the enhanced MDA levels and decreased SOD, CAT, and GSH activities in mice.

### Enhanced autophagy in the kidneys of chronic intermittent hypoxia and sleep-deprived mice

**Figure 3.**
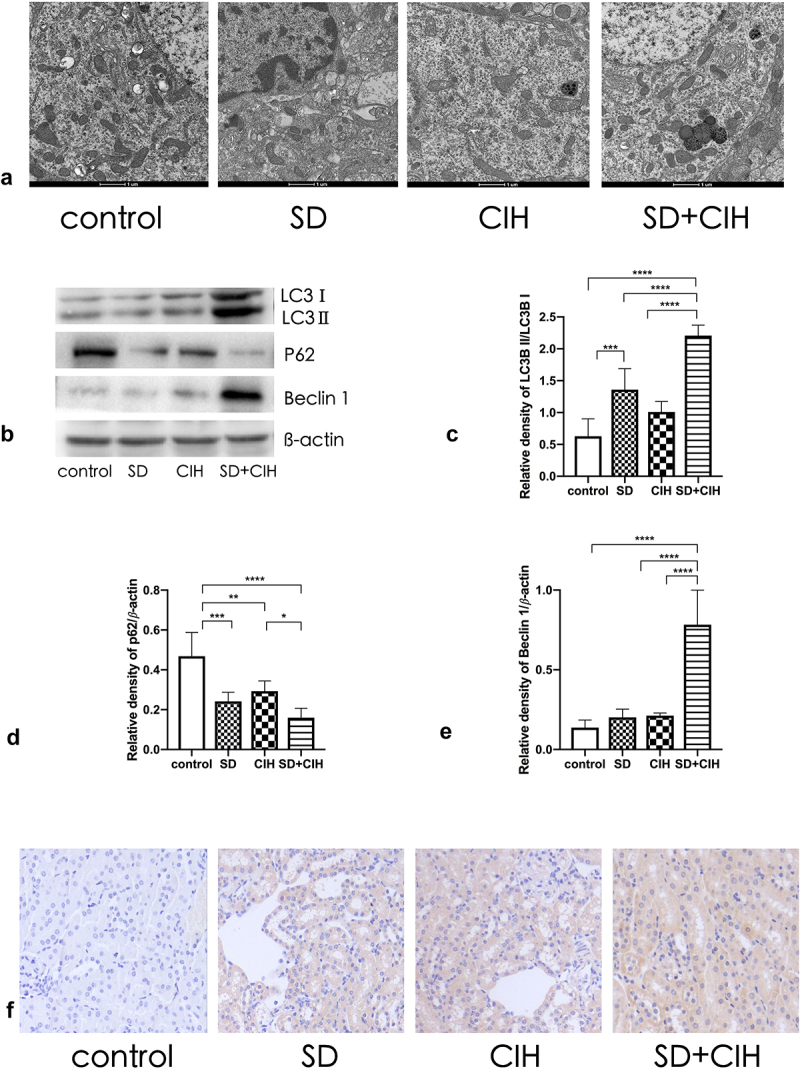
Enhanced autophagy in the kidneys of chronic intermittent hypoxia and sleep-deprived mice. (a) Representative electron microscope diagram of the kidney after different processing (1 μm); (B, C, D, E) LC3, P62, and Beclin 1 expression levels are determined by immunoblotting. Data are expressed as mean ± standard deviation (n = 6) and are analyzed by one-way analysis of variance. (f) Immunohistochemistry (IHC) images of LC3 degree of staining of mouse kidney (original magnification, × 20). *p < 0.05; **p < 0.01; ***p < 0.001; ****p < 0.0001.

Next, we investigated the role of autophagy in SD/CIH-induced kidney injury. To understand the effect of SD/CIH on the microscopic substructure of the kidney, we observed the kidney using transmission electron microscopy. Compared to the control, SD, and CIH groups, the nucleus ruptured, and the morphological structure of the cells was unclear in the SD+CIH group (Figure 3(a)). Western blot analysis revealed that the SD+CIH group showed increased LC3 and Beclin 1 protein expression and reduced P62 protein expression compared with the control, SD, and CIH groups (Figure 3(b,e)). Compared to the control, SD, and CIH groups, the SD+CIH group had a higher degree of LC3 staining (Figure 3(f)).

### Rapamycin ameliorates renal damage mediated by autophagy

**Figure 4.**
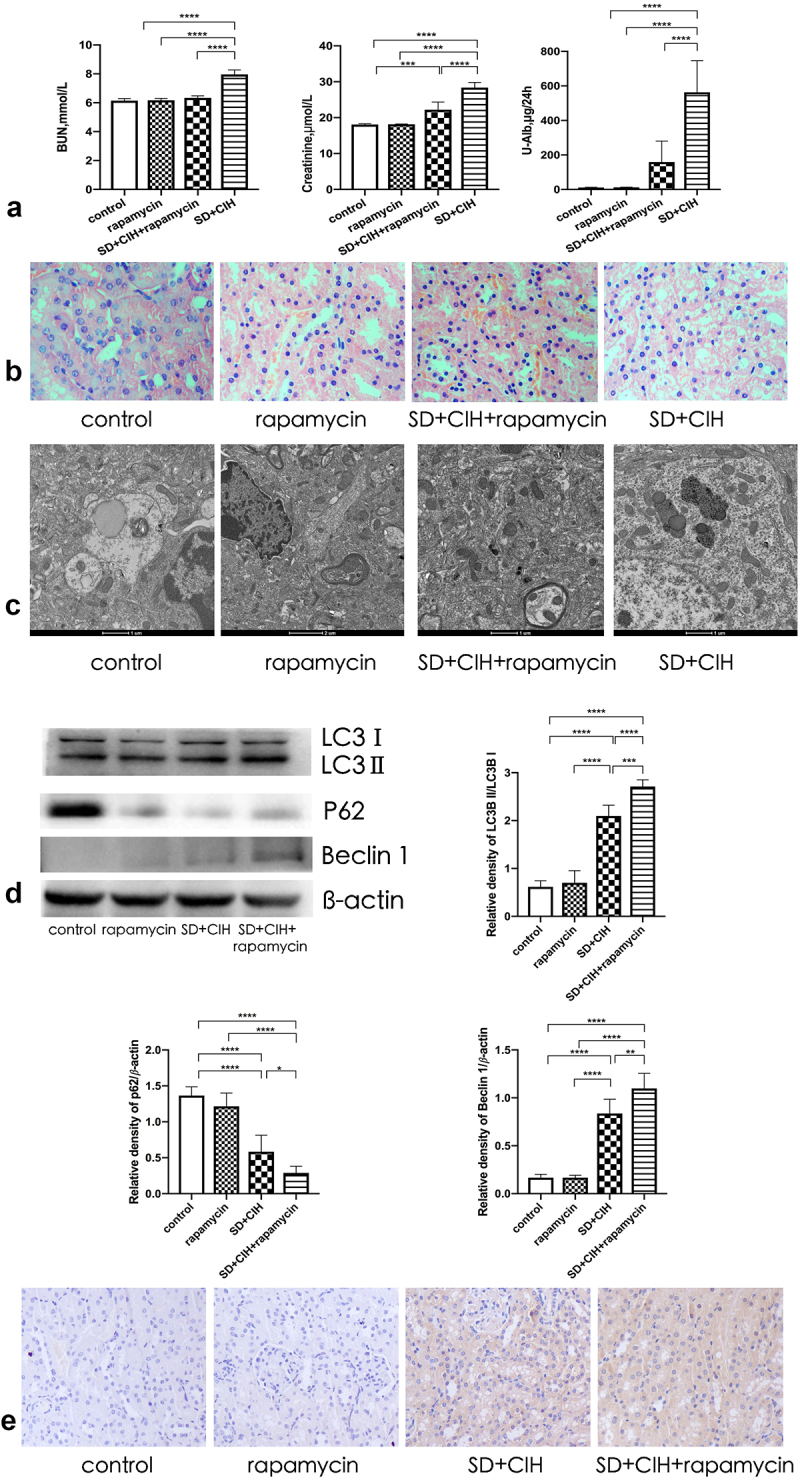
Rapamycin ameliorates renal damage mediated by autophagy. (a) Blood urea nitrogen (BUN), creatinine, urinary albumin (U-Alb); (b) Representative images of hematoxylin and eosin (HE)-stained kidney tissues in four different groups (original magnification, × 40); (c) Representative electron microscope diagram of the kidney after different processing (1 μm); (d) LC3, P62, and Beclin 1 expression levels are determined using immunoblotting. Data are expressed as mean ± standard deviation (n = 6) and are analyzed by one-way analysis of variance. (e) Immunohistochemistry (IHC) images of LC3 degree of staining of mouse kidney (original magnification, × 20). *p < 0.05; **p < 0.01; ***p < 0.001; ****p < 0.0001.

Treatment with rapamycin, an agonist of autophagy, for 3 successive weeks protected mice against kidney injury. Compared to the SD+CIH group, BUN, creatinine, and U-Alb levels were decreased in the SD+CIH+rapamycin group (Figure 4(a)). The structure and histology of the kidney after rapamycin treatment were well preserved, including decreased nucleus abscission and inflammatory cell infiltration (Figure 4(b,c)). Thus, treatment with rapamycin significantly ameliorated kidney damage. Compared to the SD+CIH group, Western blot analysis revealed that the SD+CIH+rapamycin group showed increased LC3 and Beclin 1 protein expression and reduced P62 protein expression (Figure 4(d)), and it had a higher degree of LC3 staining than the other groups (Figure 4(e)).

### Rapamycin ameliorates renal damage mediated by inhibiting autophagy-dependent mTOR/NLRP3 signaling pathway

**Figure 5.**
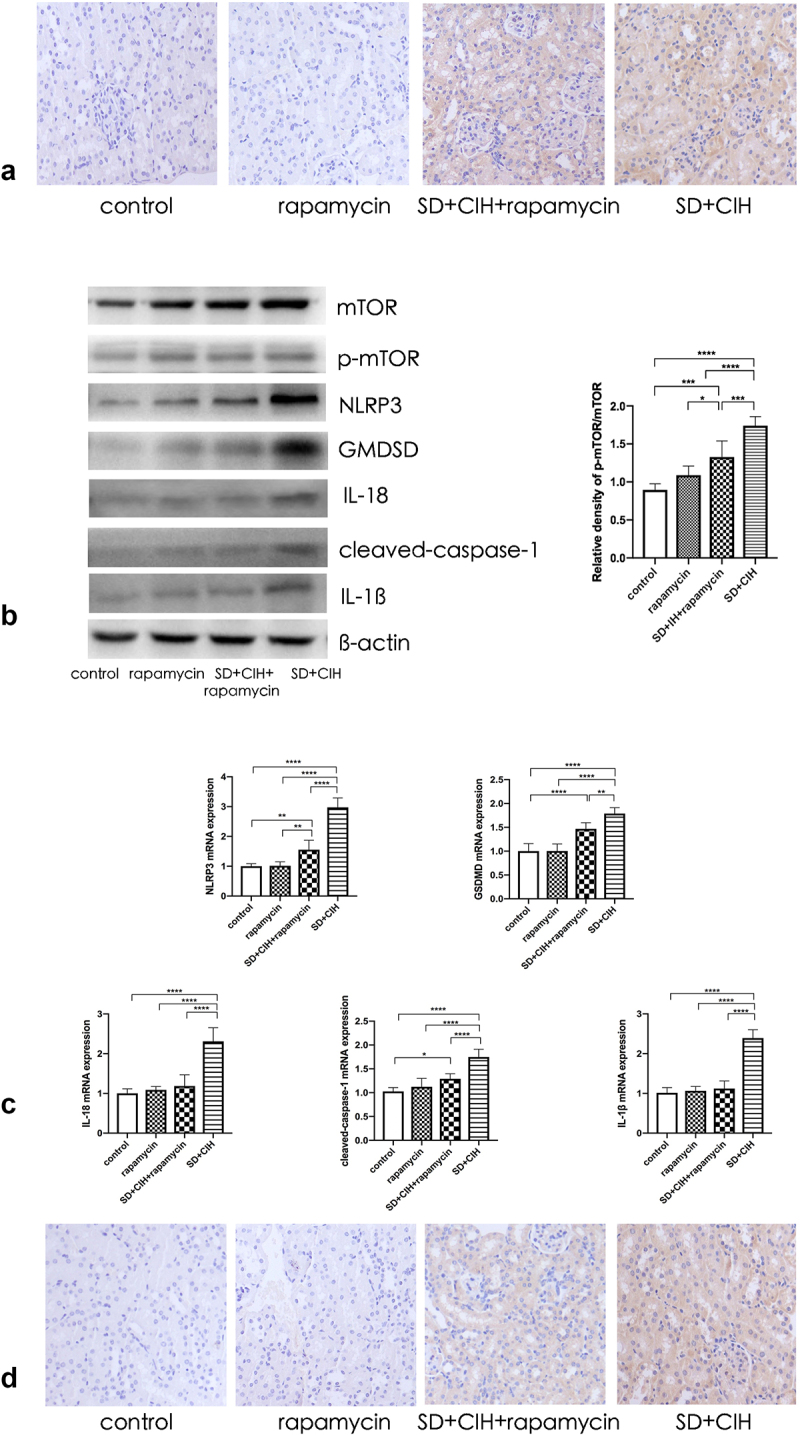
Rapamycin ameliorates renal damage mediated by inhibiting the autophagy-dependent mTOR/NLRP3 signaling pathway. (a) Immunohistochemistry (IHC) images of mTOR degree of staining of mouse kidney (original magnification, × 20); (b) Kidney mTOR, p-mTOR, NLRP3, GMDSD, IL-18, IL-1β, and cleaved-caspase-1 protein levels in mice are determined using immunoblotting; (c) Kidney mTOR, p-mTOR, NLRP3, GMDSD, IL-18, IL-1β, and cleaved-caspase-1 mRNA levels in mice are determined using reverse transcriptase-polymerase chain reaction (RT-PCR). Data are expressed as mean ± standard deviation (n = 6) and are analyzed by one-way analysis of variance. (d) Immunohistochemistry (IHC) images of NLRP3 degree of staining of mouse kidney (original magnification, × 20). *p < 0.05; **p < 0.01; ***p < 0.001; ****p < 0.0001.

The autophagy marker mTOR was increased in the kidneys of SD+CIH-exposed mice (Figure 5(a,b)). The NLRP3, GMDSD, IL-18, IL-1β, and cleaved-caspase-1 protein levels were higher in the SD+CIH group than in the SD+CIH+rapamycin group (Figure 5(b)), and NLRP3, GMDSD, IL-18, IL-1β, and cleaved-caspase-1 mRNA levels were higher in the SD+CIH group than in the SD+CIH+rapamycin group. After rapamycin treatment, pyroptosis was suppressed (Figure 5(c)). Compared to the SD+CIH+rapamycin group, the SD+CIH group showed a higher degree of NLRP3 staining (Figure 5(d)).

## Discussion

Sleep is an important physiological demand that is regulated by sleep homeostasis and circadian rhythm. Good sleep is conducive to normal body operation [24]. SD refers to a lack of sleep or a serious lack of sleep time for various reasons. Rapid eye movement is an important sleep stage to deal with the metabolic waste of brain tissue and prepare the proteins and neurotransmitters required by daytime nerve cells. Sleep in patients with OSA often has obvious rapid eye movement SD [25]. Sleep is the most important external manifestation of maintaining the circadian rhythm. When sleep problems occur, renal function is affected [26]. Intermittent hypoxia has many adverse effects on the body. After repeated hypoxia and reoxygenation, the body reacts similarly to ischemia-reperfusion injury, produces a large number of oxygen free radicals, promotes oxidative stress and inflammatory reactions, and leads to blood glucose and lipid metabolism disorders and cognitive impairment. OSA-related renal diseases can be caused by CIH-induced renal diseases [27], which could be mediated through renal iron overload, apoptosis, and increased oxidative stress [28].

Knutson et al. found that decreased sleep quality and insufficient sleep time can lead to decreased glomerular filtration rate, and decreased sleep quality can also lead to an increased protein/creatinine ratio [29]. Poor sleep status is closely associated with an increased risk of chronic kidney disease [30]. SD is an independent risk factor for early chronic kidney disease [31]. The physiological function of the kidney has a circadian rhythm [32], the excretion of urine volume, glomerular filtration rate, renal blood flow, urinary protein, electrolytes such as sodium [33], potassium, calcium, and phosphorus [34] and some metabolites depend on the circadian rhythm of the kidney. Emans et al. found that the oxygen content of the rat renal cortex and medulla has periodic changes and is high at night and low during the day, indicating that the kidney is more vulnerable to attack during sleep [35]. In the current study, the SD, CIH, and SD+CIH groups had lower body weights. Weight loss due to renal injury is due to the decline of renal function, and many wastes in the body cannot be discharged from the body, resulting in loss of appetite, insufficient energy and calorie intake, and weight loss. Additionally, after kidney injury, the body is often in a high catabolic state, due to accelerated protein catabolism and increased muscle decomposition rate, and weight loss occurs. To explore the effect of OSA on renal injury, we found that the SD+CIH group had higher BUN, creatinine, and U-Alb levels. The obvious renal injury occurred in the SD+CIH group in images of HE-stained kidney tissues, confirming that chronic intermittent hypoxia combined with SD caused renal injury in mice.

ROS are produced during cell metabolism and are important redox signals [36]. Under normal conditions, ROS are safely metabolized by cellular antioxidant mechanisms. When the generation of ROS exceeds the cellular antioxidant capacity, ROS are released during oxidative stress, and non-physiological ROS react abnormally with proteins, nucleic acids, carbohydrates, and lipids [37]. Oxidative stress is a marker of cell dysfunction and cell death. Rodrigues et al. also found that oxidative stress in Drosophila increases significantly after SD [38]. Therefore, we investigated the effect of SD/CIH on the activation of these processes. The results demonstrated that CIH+SD exposure induced oxidative damage, as evidenced by the enhanced MDA levels and decreased SOD, CAT, and GSH activities in mice. A recent study confirmed that OSA predisposed the kidneys to the adverse effects of oxidative stress and phospholipid damage [39]. Research has shown that the levels of SOD and GSH PX in the hippocampus of sleep-deprived mice decrease significantly, and MDA increases significantly, suggesting that SD leads to abnormal metabolism in the hippocampus, produces a large number of free radicals, and enhances the lipid oxidation reaction [40]. SOD is responsible for the antioxidant activity in the body. It is the only known enzyme in the body that can scavenge ROS. The decrease in SOD activity indicated that the antioxidant capacity decreased [41]. Oxidative stress leads to oxidative damage of important cellular components such as proteins, lipids, and DNA; cell death; and multiple organ damage; this will not only lead to a series of morphological changes, such as vacuolar degeneration of renal parenchymal cells and interstitial cells, but also reduce glomerular filtration function and renal tubular reabsorption function [42]. As important pathogenesis of renal injury, oxidative stress can be detected in animal models such as ischemia-reperfusion injury and sepsis [43]. Accumulating evidence indicates that CIH/SD contributes to oxidative stress and inflammation.

Autophagy refers to the degradation of cytoplasmic components in lysozyme [44]. Here, we investigated the role of autophagy in SD/CIH-induced kidney injury. To understand the effect of SD/CIH on the microscopic substructure of the kidney, we observed the kidney using transmission electron microscopy; the nuclei ruptured, and the morphological structure of the cells was unclear in the SD+CIH group. Compared to the control, SD, and CIH groups, the SD+CIH group showed a higher degree of LC3 staining, indicating that autophagy was significantly increased in the SD+CIH group, and Western blot analysis revealed that the SD+CIH group showed increased apoptosis. Generally, autophagy is an important protective mechanism of the body [45], and enhancing the autophagy process to a certain extent can appropriately reduce the oxidative damage of the body [46] and can inhibit pyroptosis; in the process of renal injury, the level of intracellular pyroptosis is an important mechanism of renal injury caused by OSA.

Rapamycin modulates multiple cellular signaling pathways in OSA. It has been shown that rapamycin can improve renal function, decrease proteinuria levels, and alleviate renal tubulointerstitial infiltration and interstitial fibrosis. Compared to the SD+CIH group, BUN, creatinine, and U-Alb levels decreased in the SD+CIH+rapamycin group, and the structure and histology of the kidney after rapamycin treatment were well preserved. Thus, treatment with rapamycin significantly ameliorated kidney damage. Compared to the SD+CIH group, Western blot analysis revealed that the SD+CIH+rapamycin group showed increased LC3 and Beclin 1 protein expression, and reduced P62 protein expression, while the SD+CIH+rapamycin group had a higher degree of LC3 staining than the other groups, indicating that rapamycin ameliorates renal damage mediated by increased autophagy. Although the level of autophagy increases, autophagy can only maintain cell homeostasis to a certain extent [47]. Simultaneously, ROS with increased concentration will attack organs and tissues in the aircraft, such as the liver and kidney, resulting in corresponding morphological and functional damage. Xu et al. found that upregulation of autophagy can reduce the level of oxidative stress in renal tissue to protect renal function [48]. In our study, treatment with rapamycin, an agonist of autophagy, for 3 successive weeks protected mice against kidney injury. In our study, the autophagy marker mTOR was increased in the kidneys of SD+CIH-exposed mice. The pyroptosis makers such as NLRP3, GMDSD, IL-18, IL-1β, and cleaved-caspase-1 protein levels and the NLRP3, GMDSD, IL-18, IL-1β, and cleaved-caspase-1 mRNA levels were higher in the SD+CIH group than in the SD+CIH+rapamycin group. Following rapamycin treatment, pyroptosis was suppressed. Compared to the SD+CIH+rapamycin group, the SD+CIH group showed a higher degree of NLRP3 staining, indicating that rapamycin ameliorated renal damage mediated by inhibition of the autophagy-dependent mTOR/NLRP3 signaling pathway. OSA can induce the activation of inflammatory factors and promote the occurrence of multi-organ inflammatory responses. The NLRP3 inflammasome is an important target of the classical focal death pathway, mediating the classical pyroptosis pathway [13], and the level of NLRP3 in patients with OSA is significantly increased. mTOR is an important target of autophagy. Inhibiting the mTOR signaling pathway can significantly reduce the levels of NLRP3 and organ damage caused by OSA. When autophagy is enhanced, it can inhibit cell focal death and reduce related organ damage through the mTOR/NLRP3 signaling pathway [49], which can be used as a potential intervention target to increase autophagy and reduce cell death to delay renal injury [14].

## Conclusion

**Figure 6.**
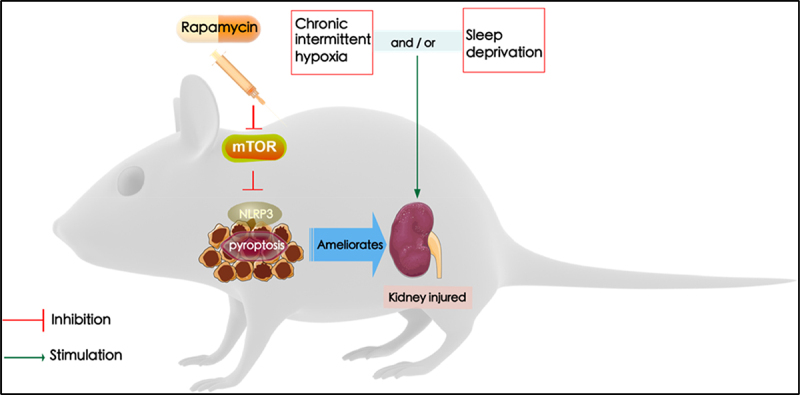
The kidney injured by sleep deprivation (SD) and intermittent hypoxia (IH) is attributed to the activation of the NLRP3 inflammasome. In contrast, inhibition of mTOR/NLRP3 with rapamycin ameliorates chronic intermittent hypoxia and sleep deprivation-induced renal damage.

In conclusion, the present study identified the effects of rapamycin on OSA-associated renal injury. Rapamycin suppression-mediated effects were dependent on its correlation with the mTOR/NLRP3 signaling pathway, consequently resulting in the attenuation of renal injury, and NLRP3 suppression-mediated effects were dependent on its correlation with the activation of mTOR, consequently resulting in the attenuation of renal injury (Figure 6). Our findings suggest that modulation of mTOR/NLRP3 pathways by rapamycin could aid in the identification of novel therapeutic strategies for the treatment of renal injury with OSA.

## Supplementary Material

Supplemental Material

## References

[1] Bhaskar S, Hemavathy D, Prasad S. Prevalence of chronic insomnia in adult patients and its correlation with medical comorbidities[J]. J Family Med Prim Care. 2016;5(4):780–784. 28348990 10.4103/2249-4863.201153 PMC5353813

[2] Coussens S, Baumert M, Kohler M, et al. Movement distribution: a new measure of sleep fragmentation in children with upper airway obstruction[J]. Sleep. 2014;37(12):2025–2034. 25325486 10.5665/sleep.4264 PMC4548508

[3] Gottlieb DJ, Ellenbogen JM, Bianchi MT, et al. Sleep deficiency and motor vehicle crash risk in the general population: a prospective cohort study[J]. BMC Med. 2018;16(1):44. 29554902 10.1186/s12916-018-1025-7 PMC5859531

[4] Scammell TE, Arrigoni E, Lipton JO. Neural Circuitry of Wakefulness and Sleep[J]. Neuron. 2017;93(4):747–765. 28231463 10.1016/j.neuron.2017.01.014 PMC5325713

[5] Marrone O, Cibella F, Pepin JL, et al. Fixed But Not Autoadjusting Positive Airway Pressure Attenuates the Time-dependent Decline in Glomerular Filtration Rate in Patients With OSA[J]. Chest. 2018;154(2):326–334. 29698721 10.1016/j.chest.2018.04.020

[6] Dou L, Lan H, Reynolds DJ, et al. Association between Obstructive Sleep Apnea and Acute Kidney Injury in Critically Ill Patients: a Propensity-Matched Study[J]. Nephron. 2017;135(2):137–146. 27951537 10.1159/000453367

[7] Cheungpasitporn W, Thongprayoon C, Gonzalez-Suarez ML, et al. The effects of short sleep duration on proteinuria and chronic kidney disease: a systematic review and meta-analysis[J]. Nephrol Dial Transplant. 2017;32(6):991–996. 27190375 10.1093/ndt/gfw072

[8] Manchanda S, Singh H, Kaur T, et al. Low-grade neuroinflammation due to chronic sleep deprivation results in anxiety and learning and memory impairments[J]. Mol Cell Biochem. 2018;449(1–2):63–72. 29549603 10.1007/s11010-018-3343-7

[9] Dennis JM, Witting PK. Protective Role for Antioxidants in Acute Kidney Disease[J]. Nutrients. 2017;9(7):718. 10.3390/nu9070718 PMC5537833 28686196

[10] Sureshbabu A, Ryter SW, Choi ME. Oxidative stress and autophagy: crucial modulators of kidney injury[J]. Redox Biol. 2015;4:208–214. 25613291 10.1016/j.redox.2015.01.001 PMC4803795

[11] Sanchez-de-la-torre M, Sanchez-de-la-torre A, Bertran S, et al. Effect of obstructive sleep apnoea and its treatment with continuous positive airway pressure on the prevalence of cardiovascular events in patients with acute coronary syndrome (ISAACC study): a randomised controlled trial[J]. Lancet Respir Med. 2020;8(4):359–367. 31839558 10.1016/S2213-2600(19)30271-1

[12] Yang Y, Zhang Y, Gao J, et al. Pyrethrum extract induces oxidative DNA damage and AMPK/mTOR-mediated autophagy in SH-SY5Y cells[J]. Sci Total Environ. 2020;740:139925. 32562985 10.1016/j.scitotenv.2020.139925

[13] Du P, Wang J, Han Y, et al. Blocking the LncRNA MALAT1/miR-224-5p/NLRP3 Axis Inhibits the Hippocampal Inflammatory Response in T2DM With OSA[J]. Front Cell Neurosci. 2020;14:97. 32477065 10.3389/fncel.2020.00097 PMC7235443

[14] Liu J, Li T, Wu H, et al. Lactobacillus rhamnosus GG strain mitigated the development of obstructive sleep apnea-induced hypertension in a high salt diet via regulating TMAO level and CD4(+) T cell induced-type I inflammation[J]. Biomed Pharmacother. 2019;112:108580. 30784906 10.1016/j.biopha.2019.01.041

[15] Zhang X, Huang F, Chen X, et al. Ginsenoside Rg3 attenuates ovariectomy-induced osteoporosis via AMPK/mTOR signaling pathway[J]. Drug Dev Res. 2020;81(7):875–884. 32898934 10.1002/ddr.21705

[16] Barata PC, Rini BI. Treatment of renal cell carcinoma: current status and future directions[J]. CA Cancer J Clin. 2017;67(6):507–524. 28961310 10.3322/caac.21411

[17] Lock HR, Sacks SH, Robson MG. Rapamycin at subimmunosuppressive levels inhibits mesangial cell proliferation and extracellular matrix production[J]. Am J Physiol Renal Physiol. 2007;292(1):F76–81. 16896187 10.1152/ajprenal.00128.2006

[18] Kramer S, Wang-Rosenke Y, Scholl V, et al. Low-dose mTOR inhibition by rapamycin attenuates progression in anti-thy1-induced chronic glomerulosclerosis of the rat[J]. Am J Physiol Renal Physiol. 2008;294(2):F440–449. 18094032 10.1152/ajprenal.00379.2007

[19] Zhang H, Zhou L, Zhou Y, et al. Intermittent hypoxia aggravates non-alcoholic fatty liver disease via RIPK3-dependent necroptosis-modulated Nrf2/NFkappaB signaling pathway[J]. Life Sci. 2021;285:119963. 34536498 10.1016/j.lfs.2021.119963

[20] Li Y, Zhang Y, Ji G, et al. Autophagy Triggered by Oxidative Stress Appears to Be Mediated by the AKT/mTOR Signaling Pathway in the Liver of Sleep-Deprived Rats[J]. Oxid Med Cell Longev. 2020;2020:6181630. 32148653 10.1155/2020/6181630 PMC7044486

[21] Ji D, Liu Z, Liu L, et al. Smartphone-based integrated voltammetry system for simultaneous detection of ascorbic acid, dopamine, and uric acid with graphene and gold nanoparticles modified screen-printed electrodes[J]. Biosens Bioelectron. 2018;119:55–62. 30098467 10.1016/j.bios.2018.07.074

[22] Zhou L, Tang J, Hu F, et al. Effects of different levels of TGF-beta expression and tumor cell necrosis rates in osteosarcoma on the chemotherapy resistance of osteosarcoma[J]. J Bone Oncol. 2020;23:100299. 32566472 10.1016/j.jbo.2020.100299 PMC7296333

[23] Fang YY, Luo M, Yue S, et al. 7,8-Dihydroxyflavone protects retinal ganglion cells against chronic intermittent hypoxia-induced oxidative stress damage via activation of the BDNF/TrkB signaling pathway[J]. Sleep Breath. 2021; DOI:10.1007/s11325-021-02400-5. 33993395

[24] Weber F, Dan Y. Circuit-based interrogation of sleep control[J]. Nature. 2016;538(7623):51–59. 27708309 10.1038/nature19773

[25] Joosten SA, Landry SA, Wong AM, et al. Assessing the Physiologic Endotypes Responsible for REM- and NREM-Based OSA[J]. Chest. 2021;159(5):1998–2007. 33197399 10.1016/j.chest.2020.10.080

[26] Sakaguchi Y, Shoji T, Kawabata H, et al. High prevalence of obstructive sleep apnea and its association with renal function among nondialysis chronic kidney disease patients in Japan: a cross-sectional study[J]. Clin J Am Soc Nephrol. 2011;6(5):995–1000. 21415314 10.2215/CJN.08670910 PMC3087795

[27] Zhang Y, Su X, Zou F, et al. Toll-like receptor-4 deficiency alleviates chronic intermittent hypoxia-induced renal injury, inflammation, and fibrosis[J]. Sleep Breath. 2019;23(2):503–513. 30099700 10.1007/s11325-018-1704-9

[28] Guan P, Sun ZM, Luo LF, et al. Hydrogen Gas Alleviates Chronic Intermittent Hypoxia-Induced Renal Injury through Reducing Iron Overload[J]. Molecules. 2019;24(6):1184. 10.3390/molecules24061184 PMC6471060 30917568

[29] Knutson KL, Lash J, Ricardo AC, et al. Habitual sleep and kidney function in chronic kidney disease: the Chronic Renal Insufficiency Cohort study[J]. J Sleep Res. 2018;27(2):281–289. 28643350 10.1111/jsr.12573 PMC6480301

[30] Bo Y, Yeoh EK, Guo C, et al. Sleep and the Risk of Chronic Kidney Disease: a Cohort Study[J]. J Clin Sleep Med. 2019;15(3):393–400. 30853043 10.5664/jcsm.7660 PMC6411180

[31] Casey KR. Sleep disorders in chronic kidney disease[J]. Sleep Med. 2010;11(3):231–232. 20156701 10.1016/j.sleep.2010.01.002

[32] Zhang R, Lahens NF, Ballance HI, et al. A circadian gene expression atlas in mammals: implications for biology and medicine[J]. Proc Natl Acad Sci U S A. 2014;111(45):16219–16224. 25349387 10.1073/pnas.1408886111 PMC4234565

[33] Nikolaeva S, Pradervand S, Centeno G, et al. The circadian clock modulates renal sodium handling[J]. J Am Soc Nephrol. 2012;23(6):1019–1026. 22440902 10.1681/ASN.2011080842 PMC3358761

[34] Giskeodegard GF, Davies SK, Revell VL, et al. Diurnal rhythms in the human urine metabolome during sleep and total sleep deprivation[J]. Sci Rep. 2015;5(1):14843. 26450397 10.1038/srep14843 PMC4598809

[35] Emans TW, Janssen BJ, Joles JA, et al. Circadian Rhythm in Kidney Tissue Oxygenation in the Rat[J]. Front Physiol. 2017;8:205. 28428757 10.3389/fphys.2017.00205 PMC5382217

[36] Lu Z, Imlay JA. When anaerobes encounter oxygen: mechanisms of oxygen toxicity, tolerance and defence[J]. Nat Rev Microbiol. 2021;19(12):774–785. 34183820 10.1038/s41579-021-00583-y PMC9191689

[37] Sies H, Jones DP. Reactive oxygen species (ROS) as pleiotropic physiological signalling agents[J]. Nat Rev Mol Cell Biol. 2020;21(7):363–383. 32231263 10.1038/s41580-020-0230-3

[38] Rodrigues NR, Macedo GE, Martins IK, et al. Short-term sleep deprivation with exposure to nocturnal light alters mitochondrial bioenergetics in Drosophila[J]. Free Radic Biol Med. 2018;120:395–406. 29655867 10.1016/j.freeradbiomed.2018.04.549

[39] Sivam S, Singer R, Yee BJ. Does CPAP Improve Chronic Kidney Disease in OSA?: it’s Still a Maybe[J]. Chest. 2021;159(5):1717–1718. 33965129 10.1016/j.chest.2021.01.014

[40] Alzoubi KH, Malkawi BS, Khabour OF, et al. Arbutus andrachne L. Reverses Sleep Deprivation-Induced Memory Impairments in Rats[J]. Mol Neurobiol. 2018;55(2):1150–1156. 28101814 10.1007/s12035-017-0387-8

[41] Krenzlin H, Wesp D, Schmitt J, et al. Decreased Superoxide Dismutase Concentrations (SOD) in Plasma and CSF and Increased Circulating Total Antioxidant Capacity (TAC) Are Associated with Unfavorable Neurological Outcome after Aneurysmal Subarachnoid Hemorrhage[J]. J Clin Med. 2021;10(6):1188. 33809085 10.3390/jcm10061188 PMC7999673

[42] Ratliff BB, Abdulmahdi W, Pawar R, et al. Oxidant Mechanisms in Renal Injury and Disease[J]. Antioxid Redox Signal. 2016;25(3):119–146. 26906267 10.1089/ars.2016.6665 PMC4948213

[43] Zhu YB, Zhang YP, Zhang J, et al. Evaluation of Vitamin C Supplementation on Kidney Function and Vascular Reactivity Following Renal Ischemic Injury in Mice[J]. Kidney Blood Press Res. 2016;41(4):460–470. 27415798 10.1159/000443447

[44] Strzyz P. Faulty endocytosis ENDing with autophagy[J]. Nat Rev Mol Cell Biol. 2021;22(1):1. 33244181 10.1038/s41580-020-00316-8

[45] Li YF, Ouyang SH, Tu LF, et al. Caffeine Protects Skin from Oxidative Stress-Induced Senescence through the Activation of Autophagy[J]. Theranostics. 2018;8(20):5713–5730. 30555576 10.7150/thno.28778 PMC6276298

[46] de Figueroa P L, Lotz MK, Blanco FJ, et al. Autophagy activation and protection from mitochondrial dysfunction in human chondrocytes[J]. Arthritis Rheumatol. 2015;67(4):966–976. 25605458 10.1002/art.39025 PMC4380780

[47] Liu K, Zhao Q, Liu P, et al. ATG3-dependent autophagy mediates mitochondrial homeostasis in pluripotency acquirement and maintenance[J]. Autophagy. 2016;12(11):2000–2008. 27575019 10.1080/15548627.2016.1212786 PMC5103358

[48] Xu J, Liu LQ, Xu LL, et al. Metformin alleviates renal injury in diabetic rats by inducing Sirt1/FoxO1 autophagic signal axis[J]. Clin Exp Pharmacol Physiol. 2020;47(4):599–608. 31821581 10.1111/1440-1681.13226

[49] Cosin-Roger J, Simmen S, Melhem H, et al. Hypoxia ameliorates intestinal inflammation through NLRP3/mTOR downregulation and autophagy activation[J]. Nat Commun. 2017;8(1):98. 28740109 10.1038/s41467-017-00213-3 PMC5524634

