# Supplementary material for: Rapamycin ameliorates chronic intermittent hypoxia and sleep deprivation-induced renal damage via the mammalian target of rapamycin (mTOR)/NOD-like receptor protein 3 (NLRP3) signaling pathway
Source: Bioengineered. 2022 Feb 19;13(3):5537–50. doi: 10.1080/21655979.2022.2037872 (PMC8973698; doi:10.1080/21655979.2022.2037872)
Supplement: Supplemental Material [file KBIE_A_2037872_SM3639.pdf]

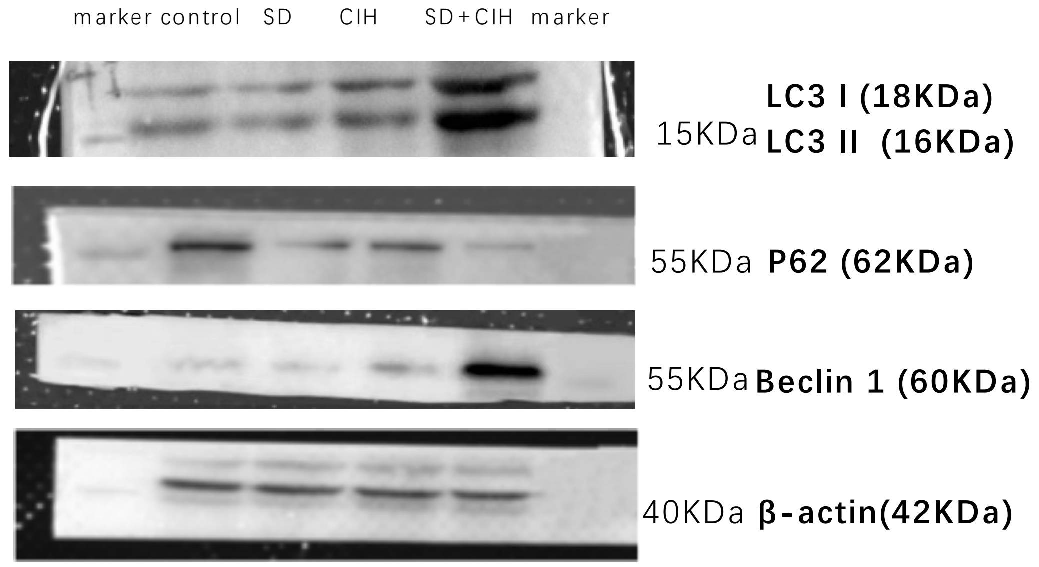

Figure 3B

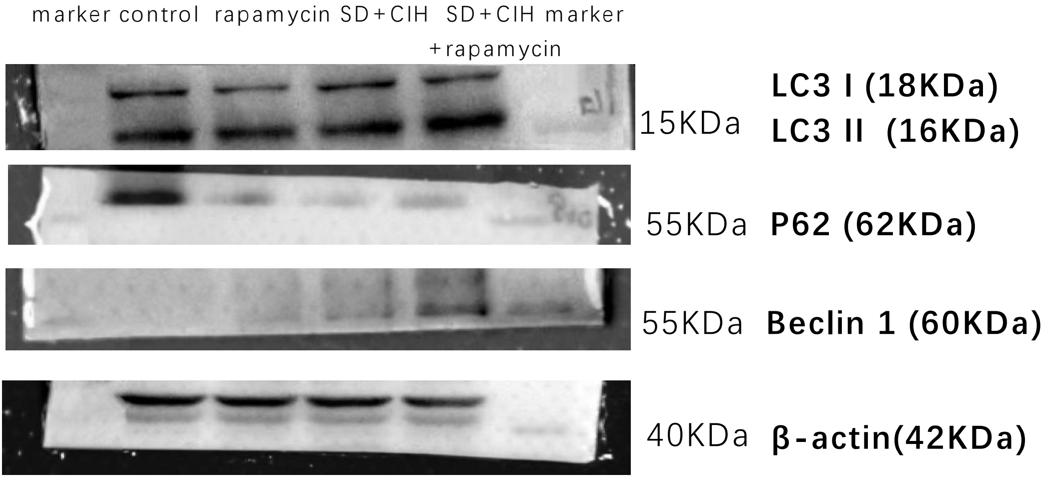

Figure 4D

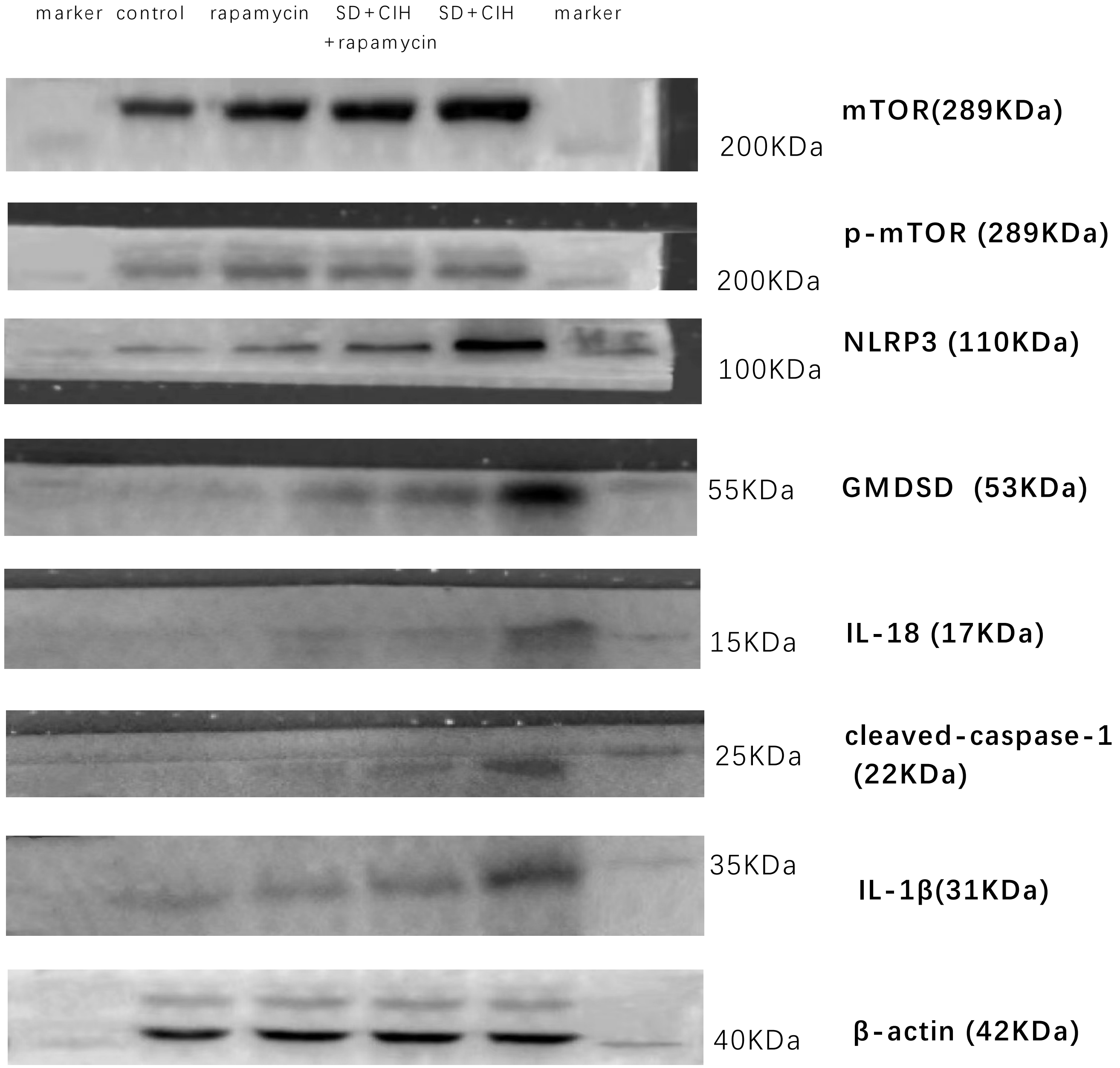

Figure 5B

Blot merged by *Image Lab*
